# Cost-effectiveness of microsurgical reconstructions in reconstructive plastic surgery within the Brazilian Public Health System: a 15-year overview (2009-2023)

**DOI:** 10.31744/einstein_journal/2026AO2003

**Published:** 2026-04-24

**Authors:** Dov Charles Goldenberg, Marcos Antonio Neves Noronha, Klaus Werner Wende, Ricardo Yugi Eri, Beatriz Peral Venet Ferreira, Lucas Andrew Jikal, Vitor Pelogi Arienzo

**Affiliations:** 1 Hospital Israelita Albert Einstein Sao Paulo SP Brazil Hospital Israelita Albert Einstein, Sao Paulo, SP, Brazil.

**Keywords:** Microsurgery, Cost-effectiveness analysis, Plastic surgery procedures, Health system, Oncology

## Abstract

**Objective:**

To evaluate the national distribution and cost-effectiveness of oncologic microsurgical reconstructions performed within the Brazilian Public Health System over a 14-year period.

**Methods:**

A retrospective analysis of DATASUS data was conducted using three procedure codes related to oncologic microsurgical reconstruction. Surgical volume was assessed by state and geographic region. Hospital reimbursement values were compared with those of less complex reconstructive procedures, and inter-state patient referral patterns were analyzed to identify trends in service centralization.

**Results:**

Between 2009 and 2023, a total of 1,902 microsurgical reconstructions were performed nationwide. The Southeast and South regions accounted for the majority of cases, with São Paulo (n=563) and Rio Grande do Sul (n=474) representing the highest-volume states. In contrast, the North and Center-West regions exhibited substantially lower availability of microsurgical services. Reimbursement analyses demonstrated that microsurgical procedures were compensated at levels comparable to those of less complex reconstructions, despite their greater technical and infrastructural requirements. Referral flow analyses revealed a pronounced concentration of patients toward a limited number of high-volume centers.

**Conclusion:**

Microsurgical reconstruction within the Brazilian Public Health System is cost-effective; however, its availability remains unevenly distributed, restricting equitable access to complex oncologic reconstructive care. Public policies focused on workforce training, capacity-building, and regional decentralization are necessary to expand access and reduce reliance on a small number of specialized centers.

## Highlights

■ 1,902 microsurgical reconstructions performed in the Brazilian Public Health System (2009-2023).
■ Southern and Southeastern regions concentrate >85% of the procedures.
■ Microsurgery is cost-effective despite its greater complexity.
■ Centralization in São Paulo indicates a need for regional expansion.

## In Brief

A national analysis of 1,902 oncologic microsurgical reconstructions in the Brazilian Public Health System (2009-2023) showed high cost-effectiveness, but marked regional inequality, with a strong concentration of procedures in the South and Southeast. Expanding the regional capacity is essential for equitable access.

## Graphical abstract

**Figure:**
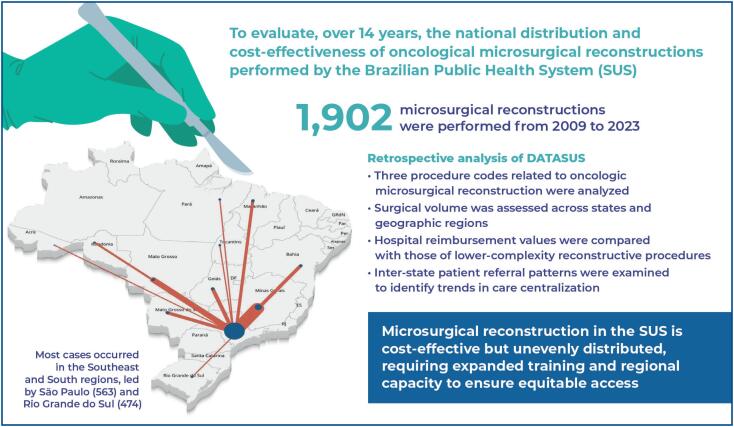

## INTRODUCTION

The importance of surgical treatments has become well established over the past two decades, with evidence suggesting that approximately 11% of disability-adjusted life years (DALYs) can be addressed through surgical care.^(1)^ Furthermore, accumulating evidence has demonstrated that surgical interventions are highly cost-effective, even when compared with well-established public health measures.^(2)^ However, access to surgical services remains unequal, particularly in low- and middle-income countries, where availability is often limited.^(3)^ Brazil reflects this pattern, with specialized centers predominantly concentrated in more developed states, especially in relation to surgical subspecialties.^(4)^ For effective health planning, it is essential to identify which surgical procedures are available in each region and to assess population access to these services, thereby enabling strategic and equitable improvements.

Plastic surgery is fundamentally based on tissue reconstruction and repair and offers a broad range of therapeutic options. Traditionally, the reconstructive ladder concept has been used, advocating for the simplest possible intervention to address tissue defects. However, Gottlieb et al. argued that the simplest approach is not always the most appropriate and that more complex interventions may sometimes be necessary to achieve superior functional and aesthetic outcomes, an approach referred to as the reconstructive elevator.^(5)^ Among the most complex procedures are microsurgical free-flap transfers, which are highly versatile for defect coverage and are considered the gold standard of reconstructive therapy.^(6)^

A major challenge to implementing the reconstructive elevator in developing countries is the cost of microsurgical procedures, which is generally higher than that of simpler reconstructive techniques. Nevertheless, recent studies indicate that microsurgical reconstructions tend to be cost-effective in the long term, as they are associated with lower donor-site morbidity, high success rates, and improved functional recovery.^(1,7-10)^

When effectively implemented, plastic surgery can have a substantial impact on cost-effectiveness. For example, based on the experience of the ReSurge group in developing countries, interventions performed during 21 surgical missions between 2014 and 2017 demonstrated high cost-effectiveness, generating an estimated savings of 9,795,384 USD.^(2)^Similarly, a study conducted at a single center within the United States healthcare system, where procedures are reimbursed through insurance, also demonstrated favorable cost-effectiveness outcomes for reconstructive microsurgery.^(5)^

A meta-analysis published in 2022 reviewed seven studies on free flap reconstructions in low-income and developing countries, analyzing 290 flaps performed in 284 patients. The analysis reported a failure rate of 3.8% (n=13; 95% confidence interval, 1.9-6.3%), a favorable outcome comparable to global microsurgical flap failure rates.^(3)^ These findings further support the cost-effectiveness and feasibility of microsurgical reconstruction, even in low-resource settings.^(11,12)^

Plastic surgery also plays a critical role in oncologic care, particularly in reconstructive flap procedures following tumor resections that expose deep anatomical structures. These interventions not only provide adequate tissue coverage but also facilitate wound healing and improve postoperative recovery and functional outcomes.^(13)^ Another important advantage of microsurgical reconstruction is its superior cost-effectiveness compared with locoregional flaps. Gao et al. demonstrated that microsurgical techniques offer significant economic and clinical benefits in the treatment of patients with early-stage head and neck cancer.^(5)^ In cases of lower limb sarcoma resection following radiotherapy, microsurgical flaps reduced wound-healing complications by 24%.^(14)^ Moreover, this approach decreases the likelihood of limb amputation, thereby preserving patients’ quality of life and reducing both financial and social costs in the postoperative period. Collectively, these findings underscore microsurgical reconstruction as an essential component of modern oncologic care, improving both functional outcomes and economic efficiency.

Despite its clinical and economic relevance, national data on the distribution and cost-effectiveness of plastic surgery procedures within the Brazilian Public Health System (SUS - *Sistema Único de Saúde*) remain limited.

## OBJECTIVE

To evaluate the national distribution and cost-effectiveness of oncologic microsurgical reconstructions performed within the Brazilian Public Health System over a 14-year period.

## METHODS

Using the public data platform TABNET from DATASUS, in combination with artificial intelligence methodologies developed by the Einstein Observatory, we analyzed detailed data on the three most relevant plastic surgery procedures used in oncologic reconstruction. The analysis was based on the following SUS procedure codes: 416080081 (reconstruction with myocutaneous flap in any oncologic area), 416080090 (microsurgical reconstruction in any oncologic area), and 416080111 (reconstruction with osteomyocutaneous flap in oncology). The study period spanned from January 2009 to December 2023. The primary focus was on the volume of microsurgical procedures in oncologic settings, as most reconstructive surgeries within the Brazilian Public Health System occur in this context. Non-microsurgical reconstructions were included exclusively for comparative purposes.

Data on hospital admissions related to these procedures were obtained from Hospitalization Authorization Forms (AIHs - *Autorizações de Internação Hospitalar*), which document hospitalizations nationwide. These forms contain information on the procedures performed, length of hospital stay, associated costs, and recorded complications. They also identify the patient's place of origin and the hospital where the procedure was performed, enabling analysis of inter-state referral patterns.

Based on this dataset, we evaluated procedure volume, financial parameters, and inter-state patient referral flows across Brazil. This analysis enabled national-level comparisons of key indicators related to plastic surgery. The study employed a retrospective, longitudinal, and ecological design. All financial values were recorded in Brazilian reais (BRL); for reference, the average exchange rate in 2023 was approximately 0.17 USD per BRL.^(15)^ The aforementioned project was not evaluated by the Research Ethics Committee (CEP) because it used publicly available data from DATASUS and TabNet; therefore, there is no letter of approval from the CEP. The project was only registered in the institutional Project Management System (SGPP), under number 6089-24.

## RESULTS

During the study period, a total of 1,902 microsurgical procedures were performed nationwide. The five states with the highest volumes were São Paulo (n=563), Rio Grande do Sul (n=474), Paraná (n=295), Santa Catarina (n=139), and Rio de Janeiro (n=103) (Table 1). In contrast, the states with the lowest numbers of procedures were Roraima (n=0), Amapá (n=1), Rondônia (n=2), Rio Grande do Norte (n=2), and Paraíba (n=2) (Table 1). At the national level, the annual number of microsurgical procedures peaked in 2013, followed by a decline until 2015, relative stability thereafter, a sharp decrease in 2020, and a gradual recovery in subsequent years.

**Table 1:** Data showing the total number of studied procedures for each Brazilian state in this respective year

	2009	2010	2011	2012	2013	2014	2015	2016	2017	2018	2019	2020	2021	2022	2023	Total
RR	0	0	0	0	0	0	0	0	0	0	0	0	0	0	0	0
AP	1	0	0	0	0	0	0	0	0	0	0	0	0	0	0	1
PB	0	0	0	0	0	0	0	0	0	0	0	0	0	0	2	2
RN	0	0	0	1	0	0	1	0	0	0	0	0	0	0	0	2
RO	0	0	0	0	0	0	0	0	0	1	0	0	0	0	1	2
AM	1	0	1	0	1	0	0	0	0	0	0	0	0	0	0	3
MS	0	0	0	0	0	0	0	0	0	0	0	0	2	0	1	3
PI	0	0	0	0	1	0	0	1	0	0	0	0	0	0	3	5
ES	0	0	1	0	1	0	1	0	0	0	0	0	1	0	2	6
MA	0	0	0	0	2	1	0	0	21		0	0	0	0	0	6
GO	0	2	2	0	1	0	11		0	0	0	0	0	0	0	7
SE	0	0	0	0	0	0	0	0	0	1	0	1	0	0	5	7
MT	0	0	0	0	0	0	0	0	0	7	1	0	0	0	0	8
PA	0	0	0	1	0	0	0	0	1	0	3	1	0	2	0	8
AC	1	1	0	0	1	2	0	2	0	0	0	0	1	1	0	9
TO	2	1	0	3	0	2	1	1	2	0	0	0	0	0	0	12
AL	2	0	0	1	4	4	5	0	0	0	0	0	0	0	0	16
DF	0	0	0	0	0	4	0	0	1	2	4	1	1	1	3	17
PE	2	1	0	0	3	6	1	4	2	1	0	1	1	0	0	22
BA	2	4	2	0	2	4	2	1	1	2	1	0	0	5	9	35
MG	2	2	6	4	8	4	5	11	7	5	4	2	4	5	2	71
CE	0	0	52	25	0	1	0	1	0	1	0	0	2	0	4	86
RJ	6	5	7	6	7	10	16	7	6	9	12	2	5	2	3	103
SC	6	6	2	14	22	4	6	16	8	5	9	9	9	14	9	139
PR	14	14	14	13	19	7	9	7	18	35	30	22	28	34	31	295
RS	37	39	58	67	69	82	42	22	14	6	3	6	5	5	19	474
SP	33	52	54	44	74	54	26	37	29	39	34	23	20	24	20	563
Total	109	127	199	179	215	185	116	111	91	115	101	68	79	93	114	1,902

When stratified by geographic region, the South and Southeast accounted for the highest numbers of microsurgical reconstructions, with 908 and 743 procedures performed over the 14-year period, respectively. In contrast, the Center-West and North regions had the lowest volumes, with only 33 and 35 procedures, respectively (Table 2).

**Table 2:** Number of procedures with associated expenses

Brazil Regions	Number of procedures	Hospital expenses	Professional expenses	Total expenses	Expenses/year	Expenses/procedures
Norte	35	100.946	34.836	135.782	9.698,71	3.879,48
Nordeste	179	696.422	192.488	888.910	63.493,57	4.965,97
Centro-Oeste	33	112.938	37.516	150.454	10.746,71	4.559,21
Sudeste	718	2.683.624	837.969	3.521.594	251.442,43	4.904,72
Sul	908	2.877.463	958.203	3.835.667	273.976,21	4.224,30

As shown in table 2, the South region not only demonstrated the highest average annual investment but also performed the greatest number of procedures nationwide. Furthermore, it exhibited the second-lowest average cost per procedure. In contrast, the Center-West region showed a lower surgical volume and the third-highest average cost per procedure. These findings reveal a pronounced disparity in microsurgical reconstruction availability between the South and Southeast regions and the North and Center-West regions.

**Figure 1:**
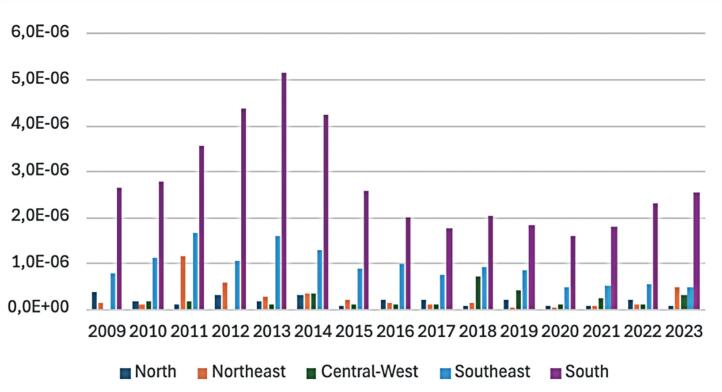
Microsurgery per million inhabitants

Figure 1 presents expenditures per million inhabitants and demonstrates substantially higher spending in the South region, followed by the Southeast. These patterns are consistent with the higher number of procedures performed in these regions. Conversely, regions with fewer procedures exhibited lower per capita expenditures.

**Figure 2:**
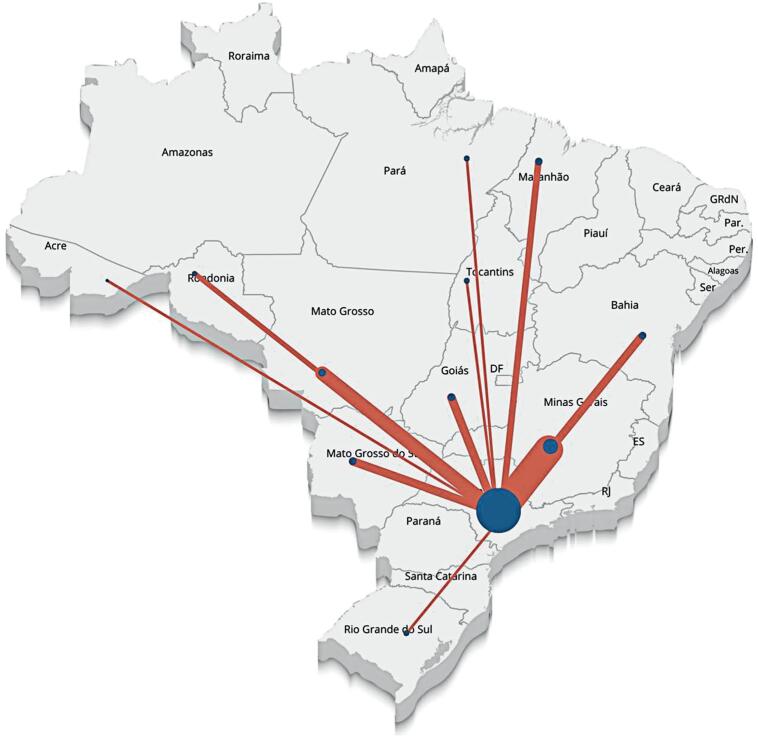
Referral flow of microsurgical reconstructions in an oncological context

Finally, figure 2 illustrates inter-state referral flows for oncologic microsurgical procedures. Orange lines represent referral connections between states, while blue circles indicate the volume of surgeries performed. A high volume of referrals from multiple states to São Paulo is evident, highlighting its role as a national reference center for oncologic microsurgical reconstruction.

## DISCUSSION

Disparities were identified in the dataset, likely reflecting irregularities in data entry and reporting within the system, with values that often failed to follow a consistent chronological trend. For example, in the North region of Brazil, five surgeries using procedure code 416080111 (microsurgical flap) were recorded in 2009, whereas only one such procedure was reported in 2023, occurring in the state of Rondônia, which had no prior records for this code. Nonetheless, an important finding is that all Brazilian states registered at least one microsurgical procedure during the study period.

São Paulo, despite being the state with the highest gross domestic product, was not consistently the leading state in procedure volume. In 2009, 2011, and 2012, Rio Grande do Sul (RS) performed the highest number of microsurgical reconstructions. In 2012, RS reached a historical peak of 67 procedures; however, this was followed by a sharp decline, with only four procedures performed in 2014, seven in 2023, and two in 2022. Such variability over time presents significant challenges for the planning and implementation of effective public health policies.

Despite inconsistencies in the dataset, marked inequality in access to microsurgical care across Brazil is evident. The South and Southeast regions account for the majority of microsurgical reconstructions nationwide. Although average costs per procedure do not differ substantially between regions, there is a clear lack of public investment in the North and Center-West regions. These findings indicate a violation of the principle of comprehensiveness established by the SUS and continue to restrict equitable patient access. The concentration of services results in most states referring patients to São Paulo, placing additional strain on the state's healthcare infrastructure and potentially limiting access for its own population. Regionalization of care is therefore essential, as it enables patients to receive treatment closer to their place of residence.

From an administrative perspective, these disparities also carry important economic implications. Reducing the need for inter-state referrals may lower financial transfers within the SUS, as Brazilian legislation mandates the transfer of funds to the receiving state whenever patients are treated outside their state of origin. As demonstrated in this study, São Paulo receives a disproportionately high number of such referrals.

In publicly funded health systems such as Brazil's, cost-effectiveness is a critical consideration when evaluating surgical procedures and hospitalizations. To date, there are no national studies comprehensively mapping the geographic distribution of microsurgical reconstructions. The present findings reveal substantial interregional disparities, with the North and Center-West regions performing very few microsurgical procedures over the study period.

Understanding the geographic distribution of microsurgical flaps is particularly important in the context of cancer, which remains one of the leading causes of morbidity and mortality worldwide. Advances in oncologic therapies have improved survival rates.^(16)^ Surgical treatment often involves tumor resection with adequate safety margins, which may require the removal of skin, muscle, and bone, resulting in large and complex defects. Although survival has improved, body image dissatisfaction has been shown to significantly affect psychosocial well-being, functional capacity, and overall quality of life among cancer survivors.^(17)^

Defects resulting from oncologic surgery are frequently extensive and may involve tissues previously exposed to radiotherapy. In such cases, traditional reconstructive options, including skin grafts and local flaps, are often insufficient or necessitate multiple staged procedures. Furthermore, local flaps may be associated with considerable donor-site morbidity, leading to additional complications. The introduction of the operating microscope provided an effective alternative in the form of free microsurgical flaps.^(18)^

Microsurgery, however, is a technically demanding subspecialty that requires prolonged training, limiting the number of qualified professionals.^(19)^ It also necessitates specialized equipment, including operating microscopes, dedicated instruments, and consumables, which increases procedural costs compared with lower levels of the reconstructive ladder.^(20)^

In many developing countries, the full potential of plastic surgery, including microsurgery, is constrained by inadequate perioperative care, limited infrastructure, and insufficient surgical training.^(7)^ Nevertheless, plastic surgery interventions in low- and middle-income countries frequently fall within the World Health Organization's threshold for being considered "highly cost-effective," defined as interventions costing less than a country's per capita gross domestic product per DALY averted. Analyses conducted by ReSurge have reported costs ranging from USD 52 to USD 11,410 per DALY averted, depending on the procedure and country, with most interventions remaining below the GDP threshold.^(2)^

Beyond cost-effectiveness, the broader economic benefits of reconstructive surgery are substantial. Human capital-based analyses estimate that plastic surgery interventions in low- and middle-income countries have generated aggregated economic gains of approximately USD 9.8 million, reflecting the potential of these procedures to restore patients’ capacity to work and resume daily activities.^(2,11)^

Reconstructive procedures using free flaps and other advanced techniques demonstrate high clinical success rates, ranging from 97% to 99% in high-income countries, and are associated with reduced long-term morbidity. However, in lower-resource settings such as Brazil, challenges including limited postoperative monitoring and resource constraints remain significant barriers.^(7)^

To address these limitations, the establishment of local training programs for surgeons and multidisciplinary teams is essential. Evidence from countries such as Vietnam and regions of Sub-Saharan Africa indicates that advanced reconstructive techniques can be successfully integrated into Public Health System, even in settings with limited infrastructure, when supported by structured training initiatives and strategic resource allocation.^(7)^

Iterative capacity-building strategies, involving repeated training missions and continuous professional education, have been shown to improve clinical outcomes and reduce long-term costs. To maximize the benefits of plastic surgery in low-resource settings, priorities should include: (1) expanding local training and developing regional centers of excellence; (2) prioritizing surgical interventions with the greatest potential impact on DALY reduction and functional restoration; and (3) incorporating low-cost technologies alongside sustainable and affordable materials.^(7)^

An important limitation of this study is its reliance on secondary data obtained from DATASUS, which depends on accurate and consistent completion of Hospitalization Authorization Forms (AIHs) by healthcare institutions nationwide. Data may be incorrectly entered, inconsistently coded, or missing altogether, particularly in regions with limited administrative capacity. This may introduce information bias through underreporting or misclassification of procedures. For example, although São Paulo reported 563 microsurgical procedures during the study period, several states reported fewer than five, and one state (Roraima) reported none. While all states recorded at least one microsurgical reconstruction between 2009 and 2023, the wide variability in annual reporting, from zero to 67 procedures per state, suggests potential underdocumentation rather than a true absence of surgical activity. This limitation may have influenced regional comparisons and the interpretation of access disparities, underscoring the need for improved data quality, transparency, and standardization within the Brazilian Public Health System.

## CONCLUSION

This study is the first to demonstrate substantial geographic inequality in the distribution of oncologic reconstructive microsurgeries in Brazil. It also identifies evidence of underreporting by certain state health systems, highlighting potential misallocation of public resources. Such discrepancies compromise inter-state financial transfers and limit optimal outcomes for patients requiring complex reconstructive procedures.

The findings reinforce the importance of microsurgical reconstruction not only for improving oncologic outcomes but also from an economic perspective, through the reduction of disability-adjusted life years and the promotion of a more economically productive population.

Furthermore, the results emphasize the need for continuous education and training programs targeting regions with lower surgical volumes. This strategy has proven effective in other countries and, given Brazil's continental size and the current concentration of procedures in the South and Southeast, expanding such initiatives represents a feasible approach to reducing regional disparities and decreasing reliance on inter-state patient referrals.

## Data Availability

The underlying content is contained within the manuscript.
